# Association of the rs3743205 variant of *DYX1C1 *with dyslexia in Chinese children

**DOI:** 10.1186/1744-9081-7-16

**Published:** 2011-05-20

**Authors:** Cadmon KP Lim, Connie SH Ho, Crystal HN Chou, Mary MY Waye

**Affiliations:** 1 Croucher Laboratory for Human Genomics, School of Biomedical Sciences, The Chinese University of Hong Kong, Shatin, N.T, Hong Kong; 2 Department of Psychology, The University of Hong Kong, Hong Kong

## Abstract

****Background**:**

Dyslexia is a learning disability that is characterized by difficulties in the acquisition of reading and spelling skills independent of intelligence, motivation or schooling. Studies of western populations have suggested that *DYX1C1 *is a candidate gene for dyslexia. In view of the different languages used in Caucasian and Chinese populations, it is therefore worthwhile to investigate whether there is an association of *DYX1C1 *in Chinese children with dyslexia.

**Method and Results:**

Eight single nucleotide polymorphisms (SNPs) were genotyped from three hundred and ninety three individuals from 131 Chinese families with two which have been reported in the literature and six tag SNPs at *DYX1C1*. Analysis for allelic and haplotypic associations was performed with the UNPHASED program and multiple testing was corrected using false discovery rates. We replicated the previously reported association of rs3743205 in Chinese children with dyslexia (*p*_*corrected *_= 0.0072). This SNP was also associated with rapid naming, phonological memory and orthographic skills in quantitative trait analysis.

**Conclusion:**

Our findings suggest that *DYX1C1 *is associated with dyslexia in people of Chinese ethnicity in Hong Kong.

## Introduction

Developmental dyslexia (DD) is a learning disability that is characterized by difficulties in the acquisition of reading and spelling skills independent of intelligence, motivation or schooling. It is the most common form of learning disability (about 80% of learning disabilities is due to dyslexia) and affects about 5-10% of school children worldwide [1,2]. Studies delineating genetic factors in developmental dyslexia have identified several putative loci (*DYX1 - DYX9*) and candidate genes (*KIAA0319, DYX1C1, DCDC2 and ROBO1*) [3]. Recently, associations with the *MRPL19/C2ORF3 *genes of *DYX3 *locus, *KIAA0319L *of *DYX8 *locus and *GRIN2B *gene have also been reported [4-6].

*DYX1C1 *is the first candidate susceptibility gene of dyslexia to be identified. A cytogenetic study revealed that two chromosome translocations [t(2;15)(q11;q21) and t(2;15)(q13;q22)] in the *DYX1 *locus co-segregated with dyslexia [7]. Taipale *et al*. [8] confirmed these translocations in another dyslexic cohort, and further reported two functional variants -3G/A (rs3743205) and 1249G/T in *DYX1C1 *associated with dyslexia. Rare variant -3A was proposed to be able to alter the *Elk-1 *transcription factor binding site and affect translation initiation, while 1249T caused a nucleotide transversion to result in a truncated protein [8].

The association of this same variant -3A has been shown to be a quantitative trait of short-term memory but it has not been shown to be associated with categorical DD [9]. Positive findings were also found in other Caucasian population cohorts but biased transmission was shown in other polymorphisms and in the common allele of -3G/A or 1249G/T [10-12]. Wigg *et al*. [10] reported significant association for the common -3G allele with reading-related phenotypes in single marker analysis and biased transmission of rs11629841 and the common haplotype of 3G/1249G in categorical DD. In Scerri's study [11], a marginally significant association was shown between the common allele (1249G) and common haplotype of the two markers (-3G/1249G) and poorer performance for the phenotypic measure of orthographic coding choice (OC-choice). Dahdouh *et al*. [12] only showed a common haplotype (G/G/G) of three markers (rs3743205/rs3743204/rs600753) to be associated in a female subgroup and the haplotype was associated with short-term memory in quantitative trait analyses, although no associations have been found with DD. In addition, other studies have also reported negative associations [13-16].

Despite these inconsistent findings, DYX1C1 has been shown to play a molecular role in brain development. Knocking down the function of *DYX1C1 *using small interfering RNA (siRNA) resulted in disruption of normal neuronal migration in the developing neocortex of embryonic rat, which could be reversed by the concurrent overexpression of *DYX1C1 *[17]. Disruption of DYX1C1 also impaired auditory processing and spatial learning in rodent models [18]. Furthermore, targeted knock down of other dyslexia susceptibility candidate genes (such as *KIAA0319 *and *DCDC2*) resulted in similar patterns of neuronal migration [19,20].

To elucidate the role of DYX1C1 in neuronal migration, its interacting protein partners were investigated. Three transcriptional factors sTFII-I, SFPQ and PARP1 bind to the promoter region of *DYX1C1 *and regulate its expression [21]. The electrophoretic mobility shift assay results suggested they trans-activate the allele -3G of rs3743205 and the binding was weak in the presence of the -3A allele. In addition, two estrogen receptors (ERs), alpha (ERa) and beta (ERb) bind to the p23 domain in the N-terminus of *DYX1C1 *[22], while heat shock proteins Hsp70 and Hsp90 bind to the TPR domains in its C-terminus [23]. In fact, over-expression of DYX1C1 affects ERa and ERb levels in a dose-dependent manner [22]. Most importantly, the functional roles of ER and its ligand (estradiol) on brain development [24], synaptic plasticity/cognition, neuroprotection [25], and memory and learning [26] have been strongly supported by extensive reviews.

To our knowledge, all current association studies on DD were performed in Caucasian populations, with no information available for non-Caucasian dyslexic cohorts. As the prevalence rate of development dyslexia in Hong Kong Chinese school-aged children was estimated to be between 9.7% and 12.6%, similar to the rate in Caucasian populations [27], study of the genetic component of dyslexia in Chinese is necessarily important. The Chinese language is known to be substantially different from Western languages, being logographic and morphosyllabic rather than being based on an alphabet [28]. Moreover, orthographic (rather than phonological) deficits were found to be the main problem for Chinese people with dyslexia, in contrast to Caucasians [29,30]. fMRI studies of Chinese people with dyslexia also revealed different biological abnormalities in their brains [31,32]. We hypothesize that Chinese people with dyslexia may be influenced by risk alleles in *DYX1C1*, and we investigated this through genotyping eight genetic variants in 393 individuals from 131 Chinese families with dyslexia.

## Materials and methods

### Subjects

In total, 393 individuals from 131 Chinese families were recruited with informed consent. This study was approved by the ethical committee of The Chinese University of Hong Kong. Each family consisted of one dyslexic child, with a total of 95 males and 36 females, aged between 5 and 16 years (mean = 8.68 ± 2.06 years). They were diagnosed as DD using the Hong Kong Test of Specific Learning Difficulties in Reading and Writing (HKT-SpLD) [33] and referred by the local education authority, child assessment centres, and a parent association. The HKT-SpLD battery consisted of 12 subtests. The subtest are broken down into three literacy tests, which are Chinese Word Reading, One-minute Reading and Chinese Word Dictation, and one rapid naming test, where subjects were asked to name digits, colours and pictures. Two subtests are phonological awareness which tests the subjects' awareness of onset and rhymes of Chinese words, and three phonological memory subtests where subjects are asked to repeat orally the syllables presented to them from a tape recorder. The final three subtests are a test of orthographic skills. This consists of 70 simple Chinese integrated characters and Arabic numbers. Half of them were left/right reversed and the subjects were asked to cross out all items with an incorrect orientation.

These 12 subtests were combined to yield five composite scores in the domains of literacy, phonological awareness, phonological memory, rapid naming and orthographic skills. The sample characteristics of these phenotypic measures are shown in table 1. To be classified as children with dyslexia, their literacy composite score and at least one cognitive composite score had to be at least one standard deviation (SD = 3) below the means (mean = 10) of their respective ages in the HKT-SpLD (cutoff score = 7). Participants in the dyslexic group fulfilled this diagnostic criterion and all of the subjects showed a normal intelligence on Raven's Standard Progressive Matrices (with IQs of 85 or above).

**Table 1:** Descriptive statistics of the HKT-SpLD subtests in the samples.

Composite Tests	Sub-tests	Mean Scaled Scores (± SD)*
Literacy		
	Chinese Word Reading	5.36 (2.18)
	One Minute Reading	5.52 (1.93)
	Chinese Word Dictation	4.27 (1.89)

Rapid Naming	Digit Rapid Naming	5.08 (2.98)

Phonological Awareness		
	Rhyme Detection	9.18 (3.11)
	Onset Detection	9.03 (3.04)

Phonological Memory		
	Word Repetition I	8.84 (3.55)
	Non-word Repetition	9.09 (3.64)
	Word repetition II	9.28 (3.28)

Orthographic Skills		
	Left-Right Reversal	7.31 (3.48)
	Lexical Decision	8.19 (3.19)
	Radical Position	9.66 (2.65)

*Scaled score: Mean = 10, 1 S.D. = 3.

### SNP markers selection

SNPs were selected from the DYX1C1 region spanning Chr15: 55,709,952 to 55,800,431 (Genome Reference Consortium Human Build 37, NC_000015.9). Six tag SNPs were selected using the TAGGER program as implemented in HaploView 4.1 [34] with parameters of minor allele frequency over 5% and pairwise r^2 ^threshold of 0.8, based on the population of Han Chinese genotype data generated by the HapMap project (Data Rel#22/phase II Apr 07). Two previously reported SNPs, rs3743205 (-3G > A) and rs57809907 (1249G > T), were also included in this study [8].

### DNA extraction and genotyping

Two milliliters of saliva was collected from each individual and genomic DNA was extracted using the Oragene™ DNA self-collection kit following the manufacturer's instructions (DNA Genotek, Inc., Ottawa, Canada). The concentration of the DNA was determined by Quant-iT™ DNA Assay Kit, Broad Range (Invitrogen Corporation, California, USA). Genotyping was performed using Sequenom^® ^MassARRAY^® ^iPLEX Gold assay, according to the manufacturer's instructions (Sequenom^®^, San Diego, CA, USA, http://www.sequenom.com). Briefly, 5 ng genomic DNA was first amplified to determine the genomic sequence containing the SNP. The unincorporated dNTPs in the PCR reaction was dephosphorylated by shrimp alkaline phosphatase treatment. This is followed by the iPLEX primer extension reaction to generate allele-specific extension products of different mass. The extension products were cleaned using SpectroClean resin and then dispensed onto SpectroCHIP bioarray. The products were detected using MALDI-TOF mass spectrometry and results were analyzed using SpectroTYPER software. Markers were checked for Mendelian inconsistencies and tests of Hardy-Weinberg equilibrium using Pedstats [35].

### Statistical analyses

Family-based and haplotype association analyses were performed using UNPHASED (Version 3.1.2) which employs an allelic likelihood ratio test [36]. Haplotype analysis was performed using 2- or 3- markers sliding windows method. Initially, a global analysis was performed to test for haplotypic association and then the significant haplotypes were subsequently tested for individual haplotype analysis. Haplotypes with frequencies <1% in the whole sample were excluded. The analysis option of conditioning markers was selected for testing direct association of a single marker in the significant haplotypes. Multiple testing was corrected using Qvalue software based on false discovery rates [37]. Permutation test (1000 runs) was also used to run multiple testing corrections over all tests performed in single-marker association analyses of categorical DD. Linkage disequilibrium (LD) was calculated and LD plots were generated using Haploview version 4.1 http://www.broad.mit.edu/mpg/haploview[34].

## Results

### Single marker analysis

The call rate of genotyping was least 96% and Mendelian inconsistencies made up about 0.85% of the data. Genotypes of Mendelian error were eventually excluded from the analysis. Single marker association showed that SNPs rs3743205 (p = 0.0009, OR = 0.08, 95% CI = 0.01 to 0.64) and rs4774768 (p = 0.0367, OR = 1.68, 95% CI = 1.02 to 2.76) were significantly associated with categorical DD (Table 2) in a family cohort. Only rs3743205 remained significant after multiple correction with FDR (q = 0.0072) and 1000 runs of permutation tests (adjusted p = 0.002997). Given the significant association of rs3743205 with categorical DD, quantitative trait analysis of literacy and cognitive skills was also tested (Table 3). rs3743205 was associated with literacy (one minute reading: p = 0.0087, q = 0.0289), rapid naming (p = 0.0079, q = 0.0289), phonological memory (non-word repetition: p = 0.0096, q = 0.0289) and orthographic skills (left-right reversal: p = 0.0087, q = 0.0289). The allele A of rs3743205 was under-transmitted in families with a dyslexic child.

**Table 2:** Single-marker analysis between SNPs and categorical DD.

rs Number	SNP	Position	Location	Allele	F	OR (95% CI)	Nominal p-value	FDR q-value
rs8040756	A/G	26589156	Intron 1	A*	0.135	1.43 (0.88 - 2.32)	0.1445	0.2823
				G	0.865			
rs3743205	G/A	26581087	5' UTR	G*	0.982	0.08 (0.01 - 0.64)	0.0009	0.0072
				A	0.018			
rs4255730	C/T	26578338	Intron 3	C*	0.670	0.85 (0.59 - 1.23)	0.3969	0.6202
				T	0.330			
rs692646	A/G	26557344	Intron 4	A*	0.067	2.00 (0.96 - 4.12)	0.0532	0.1385
				G	0.933			
rs692691	C/T	26551132	Intron 4	C*	0.947	0.86 (0.40 - 1.85)	0.6947	0.8455
				T	0.053			
rs2290981	A/G	26550245	Intron 4	A*	0.119	1.10 (0.60 - 2.02)	0.7575	0.8455
				G	0.881			
rs4774768	G/T	26517062	Intron 8	G*	0.189	1.68 (1.02 - 2.76)	0.0367	0.1385
				T	0.811			
rs57809907	G/T	26513439	Exon 10	T*	0.004	1.00 (0.06 - 15.99)	1.0000	0.9766
				G	0.996			

F = Allele frequency

*Reference allele

**Table 3:** Quantitative analysis of rs3743205 in HKT-SpLD tests.

Tested area	Tests	Nominal p-value	FDR (q-value)
Literacy			
	Chinese Word Reading (CWR)	0.5415	0.5445
	One Minute Reading (OMR)	***0.0087***	***0.0289***
	Chinese Word Dictation (CWD)	0.5273	0.5445
			
Rapid Naming	Digit Rapid Naming (DRN)	***0.0079***	***0.0289***
Phonological Awareness			
	Rhyme Detection (RD)	0.1105	0.2106
	Onset Detection (OD)	0.5445	0.5445
			
Phonological Memory			
	Word Repetition I (WRI)	0.0474	0.1138
	Non-word Repetition (NWR)	***0.0096***	***0.0289***
	Word repetition II (WRII)	0.1229	0.2106
			
Orthographic Skills			
	Left-Right Reversal (LRR)	***0.0087***	***0.0289***
	Lexical Decision (LD)	0.3002	0.4002
	Radical Position (RP)	0.1481	0.2222

### Haplotype analyses

**Figure 1:**
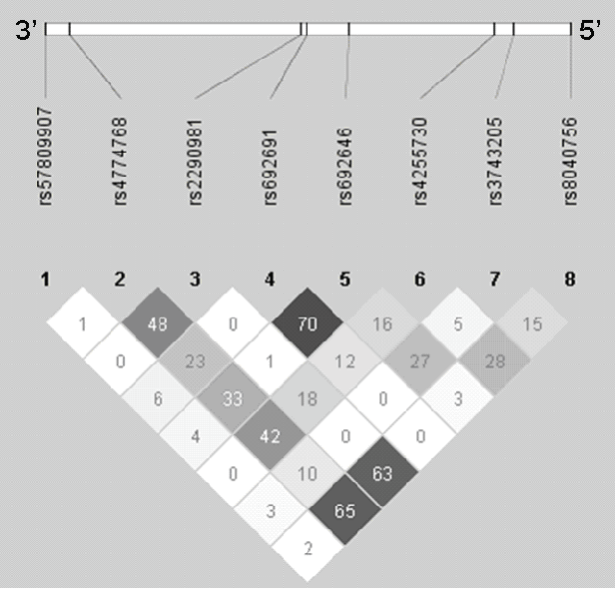
**Linkage disequilibrium plot showing the SNP markers relative location in *DYX1C1 *and their *r***^**2 **^**values**. Cells with darker shading represent highest *r*^2 ^values between SNPs.

The four haplotypes rs804075-rs3743205, rs3743205-rs4255730, rs692646-rs692691 and rs8040756-rs3743205-rs4255730 were significantly associated with categorical DD after corrections for multiple testing (Table 4). Their linkage disequilibrium (LD) structures are shown in Figure 1. Except for the rs692646-rs692691 haplotype (*r*^2 ^= 0.702 between the two SNPs), all other haplotypes consisted of SNPs in low LD (*r*^2 ^< 0.5). Individual haplotype analyses revealed the associated alleles in each haplotype: rs804075-rs3743205 (A-A, p = 0.0005), rs3743205-rs4255730 (A-T, p = 0.0039), rs692646-rs692691 (A-C, p = 0.0067) and rs8040756-rs3743205-rs4255730 (A-A-T, p = 0.0009). All the haplotypes which were significantly associated with categorical DD except rs692646-rs692691 were made up of the rs3743205 and had the same allele A found in single marker analysis. Direct association of the markers with the rs3743205 haplotype was tested using conditioning markers option of the software. All haplotypes consisting of the rs3743205 became insignificant associated with categorical DD after testing using conditioning markers (rs804075-rs3743205/p = 0.7814, rs3743205-rs4255730/p = 0.7593, rs8040756-rs3743205-rs4255730/p = 0.6099). With the exception of rs692646-rs692691 which was still significant whether rs692646 (p = 0.0031) or rs692691 (p = 0.0232) was set as a conditioning marker. These significant haplotypes were subsequently tested for quantitative trait analyses (Table 5). Both rs3743205-rs4255730 and rs692646-rs692691 haplotypes were associated with skills of literacy (one minute reading), rapid naming, phonological memory (non-word repetition) and orthographic skills (left-right reversal) (0.01 <*p *< 0.03). The rs692646-rs692691 haplotype was also associated with phonological awareness (rhyme detection). However, none of the haplotypes remained significant after multiple testing corrections. There are reports of haplotype rs3743205-rs57809907 (-3G > A-1249G > T) testing in the literature, but this was not tested here in the present study because the minor allele frequency (MAF) of rs57809907 was too low (T_MAF _= 0.004) in our samples.

**Table 4:** Results of the haplotype analysis using 2- or 3-markers sliding windows.

Haplotypes			Frequency	Global tests		Individual haplotype test		Testing direct association using conditioning markers option
				p-values	FDR (q-value)	OR (95% CI)	p-values	p-values
**2-markers**	rs8040756-rs3743205	A-G	0.123	*0.0002*	*0.0033*	Ref	0.6803	0.7814
						
		A-A	0.026			NA*	0.0005	rs3743205 as conditioning marker
						
		G-G	0.851			0.93(0.54 - 1.60)	0.2568	
								
	rs3743205-rs4255730	G-C	0.659	*0.0073*	*0.0255*	Ref	0.3972	0.7593
						
		G-T	0.315			0.94(0.65 - 1.38)	0.9244	rs3743205 as conditioning marker
						
		A-T	0.026			0.09(0.01 - 0.69)	0.0039	
								
	rs4255730-rs692646			NS				
								
	rs692646-rs692691	G-C	0.924	*0.0118*	*0.0329*	Ref	0.0555	0.0031 rs692691 as conditioning marker
						
		A-C	0.021			0.10(0.01 - 0.76)	0.0067	0.0232 rs692646 as conditioning marker
						
		A-T	0.055			0.78(0.35 - 1.78)	0.6831	
								
	rs692691-rs2290981			NS				
	rs2290981-rs4774768			NS				
	rs4774768-rs57809907			NS				
								

**3-markers**	rs8040756-rs3743205-rs4255730	A-G-C	0.008	*0.0019*	*0.0131*	NA^#^		0.6099 rs3743205 as conditioning marker
						
		A-G-T	0.110			0.99(0.56 - 1.77)	0.1828	
						
		A-A-T	0.024			NA*	0.0009	
						
		G-G-C	0.654			Ref	0.3361	
						
		G-G-T	0.204			0.87(0.55 - 1.36)	0.7459	
								
	rs3743205-rs4255730-rs692646			NS				
	rs4255730-rs692646-rs692691			NS				
	rs692646-rs692691-rs2290981			NS				
	rs692691-rs2290981-rs4774768			NS				
	rs2290981-rs4774768- rs57809907			NS				
								

NS = Not significant.

Ref = Reference haplotype,

NA* = OR calculation was not performed when zero value present in contingency table

NA# = individual haplotype frequency was below 1% in the samples.

**Table 5:** The significant haplotypes tested with quantitative traits analysis.

	Literacy			Rapid Naming	Phonological Awareness		Phonological Memory			Orthographic Knowledge		
					
	CWR	OMR	CWD	DRN	RD	OD	WR	NWR	WRI	LRR	LD	RP
rs8040756-rs3743205												
rs3743205-rs4255730		0.0296 (0.2328)		0.0307 (0.2328)				0.0326 (0.2328)		0.0336 (0.2328)		
rs692646 -rs692691		0.0387 (0.2328)		0.0080 (0.2328)	0.0246 (0.2328)			0.0427 (0.2328)		0.0257 (0.2328)		
rs8040756 -rs3743205 -rs4255730												

Only the significant nominal p-values and q-values (brackets) are shown (p < 0.05).

## Discussion

In this study, we demonstrated that SNP rs3743205 was associated with categorical DD in a Chinese cohort. The common allele -3G was over-transmitted in our cohort. Haplotype analysis also showed significant association with categorical DD and most associated haplotypes contain the rs3743205 allele. However, testing for direct association of the markers in the haplotypes showed that they were mainly driven by the effect of rs3743205. Therefore, only the rs692646-rs692691 haplotype showed a combined effect of two SNPs. Taipale *et al*. [8] reported rs3743205 to be associated with categorical DD but the rarer variant A was over-transmitted in children with dyslexia. Marino *et al*. [9] found the same direction of transmission as reported by Taipale *et al*. [8] but this was only marginally significant and was only found in the -3A/1249T haplotype. Wigg *et al*. [10] reported the opposite preferential transmissions of the common alleles in the -3G/1249G haplotype but the significant associated single-marker was rs11629841. Dahdouh *et al*. [12] only reported the -3G containing haplotype G/G/G at rs3743205/rs3743204/rs600753 in female dyslexics.

Dahdouh *et al*. [12] suggested that this discrepancy of associated variant (A or G) might be due to independent mutation events at *DYX1C1*, in which the common allele G is a putative *DYX1C1*-causing mutation in Central Europeans [10,11], whereas it points to a rarer allele A in the Finnish and the Italian populations [8,9].

Over-transmission of allele G reported in this study implies the under-transmission of allele A. Concordant to our result, a molecular study showed that the A allele of rs3743205 (-3G/A) can regulate *DYX1C1 *expression [21]. Using electrophoretic mobility shift assays, Tapia-Paez *et al*. [21] showed that the A allele probe had lower binding affinity for TFII-I, a transcription factor which represses *DYX1C1 *activity. Moreover, the allele A probe demonstrated increased *DYX1C1 *expression (measured using luciferase activity) compared to the G allele probe. The results from our and Tapia-Paez *et al's *study combine to suggest that the A allele of rs3743205 may confer a protective role in the development of dyslexia rather than the G allele being a causative factor.

In addition, Massinen et al showed that DYX1C1 interact with and regulates the level of ERs in a dose-dependent manner [22]. The ERs and estradiol not only impact on normal brain development [24], but also affect neuronal migration [38]. Defective neuronal migration is a key feature of knocking down dyslexia susceptibility candidate genes [17-20]. Therefore, DYX1C1 might be linked with ERs and neuronal migration in causing dyslexia. In particular, genetic variants of *DYX1C1 *(-3G or -3A allele) might affect DYX1C1 expression and subsequently, the level of ERs. Interestingly, neuronal migration influenced by estrogen was proposed as one of the mechanisms contributing to sexually dimorphic brain characteristics [39-41]. The gender ratio of Hong Kong Chinese is 1.6 males to 2.0 females [27]. Whether boys are more likely than girls to have reading disabilities is still unclear, but this gender-related mechanism might be the cause of boys being more susceptible to developing dyslexia.

With regard to quantitative traits analyses, rs3743205 was also significantly associated with one minute reading (OMR) of literacy, digit rapid naming (DRN), non-word repetition (NWR) of phonological memory and left-right reversal (LRR) of orthographic skills in this study. In other studies, Marino *et al*. [9] have reported short-term memory (STM) in linkage disequilibrium with the rarer A allele of -3G > A and a three marker haplotype G/G/G at rs3743205/rs3743204/rs600753 associated with STM only (the subjects in this study were all female) [12]. Recently, Bates *et al *[42] first reported the association of DYX1C1 polymorphisms with normal reading ability (Regular-word, irregular-word and nonword reading and spelling as well as verbal short-term memory) in 790 Australian families. They found that rs17819126 was significantly associated with all three reading measures and irregular word spelling. There was a marginal association with rs3743204 and irregular word reading and significant association with nonword reading. Also, a measure of verbal short-term memory was significantly associated with rs685935. However, neither rs3743205 nor rs57809907 previously reported by Taipale *et al*. [8] was significantly associated with any measures in the study of Bates *et al*. [42].

In the HKT-SpLD used in this study, the one minute reading (OMR) measures Chinese word reading fluency, the digit rapid naming (DRN) reflects long term learning ability of visual-verbal associations, and non-word repetition (NWR) is defined as a form of phonological short-term memory. Therefore, OMR measured in this study may approximate the skills required for regular word reading, skills measured by DRN may be similar to the acquisition of grapheme-phoneme conversion rules required in non-word reading, and NWR is similar to the verbal short-term memory in the study of Bates *et al*. [42]. In the view of associated traits, it is reasonable to suggest that our results of quantitative traits analyses closely agree with the findings of Bates *et al*. [42] but in different variants of *DYX1C1*.

Although the significant SNPs (rs3743204, rs685935 and rs17819126) reported by Bates *et al*. [42] were not genotyped in this study, the rs8040756 and rs4774768 examined in this study were in linkage disequilibrium with rs3743204 and rs685935 respectively (Han Chinese Hapmap data: r8040756-rs3743204 *r*^2 ^= 1, rs4774768-rs685935 *r*^2 ^= 0.9). These tag SNPs (rs8040756 and rs4774768) examined in this study are supposed to capture the alleles of rs3743204 and rs685935. Moreover, rs17819126 missense variation is unique in populations of European origin as shown by the minor allele frequency based on the Hapmap data is about 9% in a Utah population, 1% in Yoruba, but only 0.5% in Japanese and 0.7% in Chinese. It is worth noting that none of the eight tag SNPs based on the Chinese population was significantly associated with DD or phenotypic traits in this study, with the exception of the previously reported rs3743205. rs3743205 was not a selected tag SNP and its allele frequency (< 0.05) is beyond the threshold of it being powerful enough to detect associations of the tag SNPs genotyped in this study. Therefore, we could not rule out the association of rs17819126 that was not captured by current tag SNP markers. In addition, these results indicate that Chinese reading-related skills are associated with rare variants of DYX1C1 in the Chinese population. Further study using markers of rare variant (MAF < 0.05) might support this finding.

When taken together, DYX1C1 is suggested to be associated with Chinese dyslexia, and Chinese literacy and cognitive skills (DRN, NWR and LRR). These cognitive skills are all important reading-related skills in readers of the Chinese language and rapid naming and orthographic deficits were characterized as the main cognitive problems in Chinese dyslexic children [30,43,44]. To the best of our knowledge, this is the first genetic study showing that *DYX1C1 *is also a candidate dyslexia susceptibility gene for Hong Kong Chinese children. Again, we have shown the genetic heterogeneity of dyslexia that different variants of DYX1C1 may be associated with dyslexia in different populations. The existence of any population- and/or language-based variant in dyslexia should be clarified in future association studies. In particular, studies of other dyslexia candidate genes in Chinese are essential to provide us with a more complete picture of the universality of genetic association in dyslexia.

## Competing interests

MW is a consultant of Genetic Centre Company Limited, Hong Kong. CL is the Laboratory Director of Multigene Diagnostics Limited, Hong Kong.

## Authors' contributions

MW designed the study and supervised the overall experimental part of the project, communicated with the Association of Specific learning disability for help with recruitment of the subjects. CH supervised gathering the reading and writing performance and development of classification schemes for the dyslexic children, and communicated with Government departments and other agencies to obtain details of the phenotypes. CC assembled and input the test scores of the dyslexic children. CL designed and performed all the genotype analyses and association analyses of risk alleles. All authors discussed the results and implications and commented on the manuscript at all stages.
